# A Metabolomic and HPLC-MS/MS Analysis of the Foliar Phenolics, Flavonoids and Coumarins of the *Fraxinus* Species Resistant and Susceptible to Emerald Ash Borer

**DOI:** 10.3390/molecules23112734

**Published:** 2018-10-23

**Authors:** Sohail S. Qazi, Domenic A. Lombardo, Mamdouh M. Abou-Zaid

**Affiliations:** 1 Natural Resources Canada, Canadian Forest Service, Great Lake Forestry Centre, Sault Ste. Marie, ON P6A 2E5, Canada; soq823@mail.usask.ca (S.S.Q.); domenic.lombardo@canada.ca (D.A.L.); 2 Department of Chemical and Biochemical Engineering, University of Western Ontario, London, ON N6A 5B9, Canada

**Keywords:** Emerald Ash Borer (*Agrilus planipennis*), HPLC-MS/MS, metabolomics, *Fraxinus* spp. phenolics, flavonoids, coumarins, lignans, secoiridoids, invasive species

## Abstract

The Emerald Ash Borer (EAB), *Agrilus planipennis*, Fairmaire, an Asian invasive alien buprestid has devastated tens of millions of ash trees (*Fraxinus* spp.) in North America. Foliar phytochemicals of the genus *Fraxinus* (Oleaceae): *Fraxinus pennsylvanica* (Green ash), *F. americana* (White ash), *F. profunda* (Bush) Bush. (Pumpkin ash), *F. quadrangulata* Michx. (Blue ash), *F. nigra* Marsh. (Black ash) and *F*. *mandshurica* (Manchurian ash) were investigated using HPLC-MS/MS and untargeted metabolomics. HPLC-MS/MS help identified 26 compounds, including phenolics, flavonoids and coumarins in varying amounts. Hydroxycoumarins, esculetin, esculin, fraxetin, fraxin, fraxidin and scopoletin were isolated from blue, black and Manchurian ashes. High-throughput metabolomics revealed 35 metabolites, including terpenes, secoiridoids and lignans. Metabolomic profiling indicated several upregulated putative compounds from Manchurian ash, especially fraxinol, ligstroside, oleuropin, matairesinol, pinoresinol glucoside, 8-hydroxypinoresinol-4-glucoside, verbenalin, hydroxytyrosol-1-*O*-glucoside, totarol and ar-artemisene. Further, dicyclomine, aphidicolin, parthenolide, famciclovir, ar-turmerone and myriocin were identified upregulated in blue ash. Principal component analysis demonstrated a clear separation between Manchurian and blue ashes from black, green, white and pumpkin ashes. The presence of defensive compounds upregulated in Manchurian ash, suggests their potential role in providing constitutive resistance to EAB, and reflects its co-evolutionary history with *A. planipennis*, where they appear to coexist in their native habitats.

## 1. Introduction

The Emerald Ash borer (EAB), *Agrilus planipennis* Fairmaire (Coleoptera: Buprestidae) is an invasive wood-borer indigenous to Asia that has caused widespread mortality of North American ash (*Fraxinus* spp.) [1]. Since its accidental introduction in North America, that can be traced back to 1990 [2], EAB infestations have been identified in twenty-five American states and two Canadian provinces. EAB larvae feed from spring to mid-summer on the phloem and outer xylem tissue, which disrupt the translocation of nutrients and water that results in the ultimate death of the tree. EAB adults emerge from larval hosts and complete one to two weeks of feeding on ash foliage before they become reproductively mature. In the majority of cases, ash trees are killed within six years of EAB infestation or detection [1].

The Genus *Fraxinus* (Oleaceae, Olive family) has sixty species, of which sixteen of them are native to North America, and amongst them four species are native to Canada [3]. In North America, the EAB is a major invasive pest threatening all of the North American species, including *Fraxinus pennsylvanica* var. subintegerrima (Vahl) Fern. (Green ash), *F. americana* L. (White ash), *F. pennsylvanica* Marsh. (Red ash), *F. pennsylvanica* var. *austini* Fern. (Northern red ash), *F. nigra* Marsh. (black ash) [1,4,5,6]. *F. profunda* Bush. (Pumpkin ash) and *F. latifolia* Benth. (Oregon ash) ashes have been reported as being seldomly attacked by EAB. However, *F. quadrangulata* Michx. (blue ash) has been reported to be somewhat resistant in comparison to green and white ashes, especially where they co-occur [7,8]. The economic and ecological impact of EAB damage is enormous and has prompted the International Union for Conservation of Nature (IUCN) to put several ash species on their watch list of critically endangered species [9], including blue ash. The economic impact of EAB in North America is in the order of billions of dollars [10,11]. On the other hand, *F. mandshurica* (Manchurian ash), which is the preferred host of EAB in China is resistant to EAB infestation, because of its co-evolutionary history.

The chemistry of the genus *Fraxinus* is highly complex [12] and the defensive response of deciduous trees to wood-boring beetle attacks has been postulated to be a combination of constitutive and induced physical and chemical defenses [13]. In this connection, studies to date on the *Fraxinus* spp. has focused on the chemistry of the phloem and bark tissue, especially on clonal cultivars (greenhouse studies) of ash species. The phloem chemistry of the Manchurian, white and green ashes revealed phenolic compounds, including monolignols, lignans, phenylethanoids and secoiridoids [14]. Hydroxycoumarins and phenylethanoids (calceolariosides A and B) were identified unique to Manchurian ash. Similarly, Cipollini and co-workers investigated the distinguishing defensive compounds from the phloem of the Manchurian ash and have identified unique contents of hydroxycoumarins (fraxin, mandshurin and fraxidin hexoside) [15]. In addition, they reported phenylethanoids (calceolariosides A and B) and significantly higher concentrations of the lignans (pinoresinol glucoside and pinoresinol) from Manchurian ash. The lignans in the genus *Fraxinus* are mainly of tetrahydrofurofuran or sesamine type [16,17] and are found in their free or glycosidic forms [12].

During an investigation on the defensive chemical response of Manchurian ash the presence of phenolics, including hydroxycoumarins and phenylethanoid glycosides (calceolariosides A and B), pinoresinol (a dihexoside) and a coumarin derivative unique to Manchurian ash were reported [18]. Furthermore, high concentration levels of esculin (hydroxycoumarin), responsible for the greater resistance of Manchurian ash relative to other North American ash species was identified [18]. On the other hand, higher accumulations of lignan (pinoresinol A) in the phloem of Manchurian ash in contrast to black ash was documented during the induced resistance from EAB [19].

Ironically, to date, the foliar chemistry of North American ash has received little or no attention, despite its importance in EAB biology, as the foliage is the only source of maturation feeding for adult EAB. This study aims to investigate the metabolomic composition of six *Fraxinus* spp. (green, Manchurian, blue, black, pumpkin and white ashes) using HPLC-MS/MS and untargeted metabolomics. In addition, we have provided a comparison of metabolites between Manchurian and blue ashes that have varying levels of resistance to EAB. To the best of our knowledge, this study represents the first report using HPLC-MS/MS combined with autonomous untargeted metabolomic profiling in examining the foliar chemistry of six *Fraxinus* spp. and in describing the potential role that defensive polyphenolic compounds may play in providing resistance to EAB.

## 2. Results and Discussion

### 2.1. HPLC-MS/MS Orbitrap Analysis

**Figure 1:**
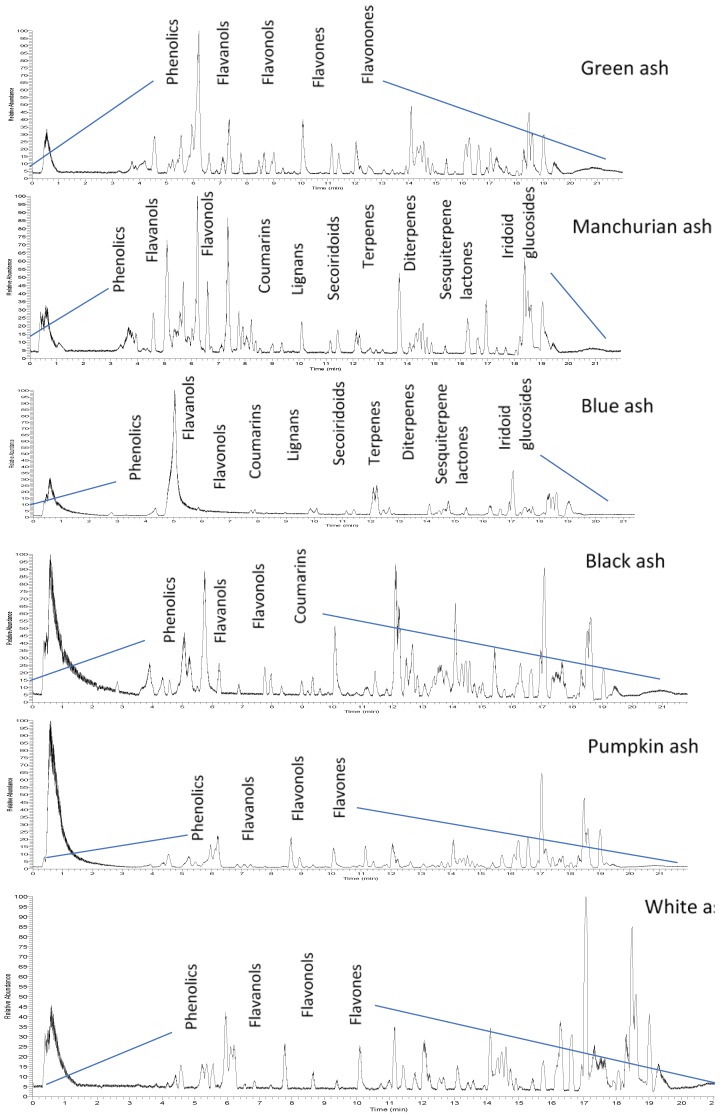
Representative HPLC-MS/MS base peak chromatograms of foliar extracts from six *Fraxinus* spp. (green, Manchurian, blue, black, pumpkin, and white ashes), revealing the diversity of separated compounds.

**Figure 2:**
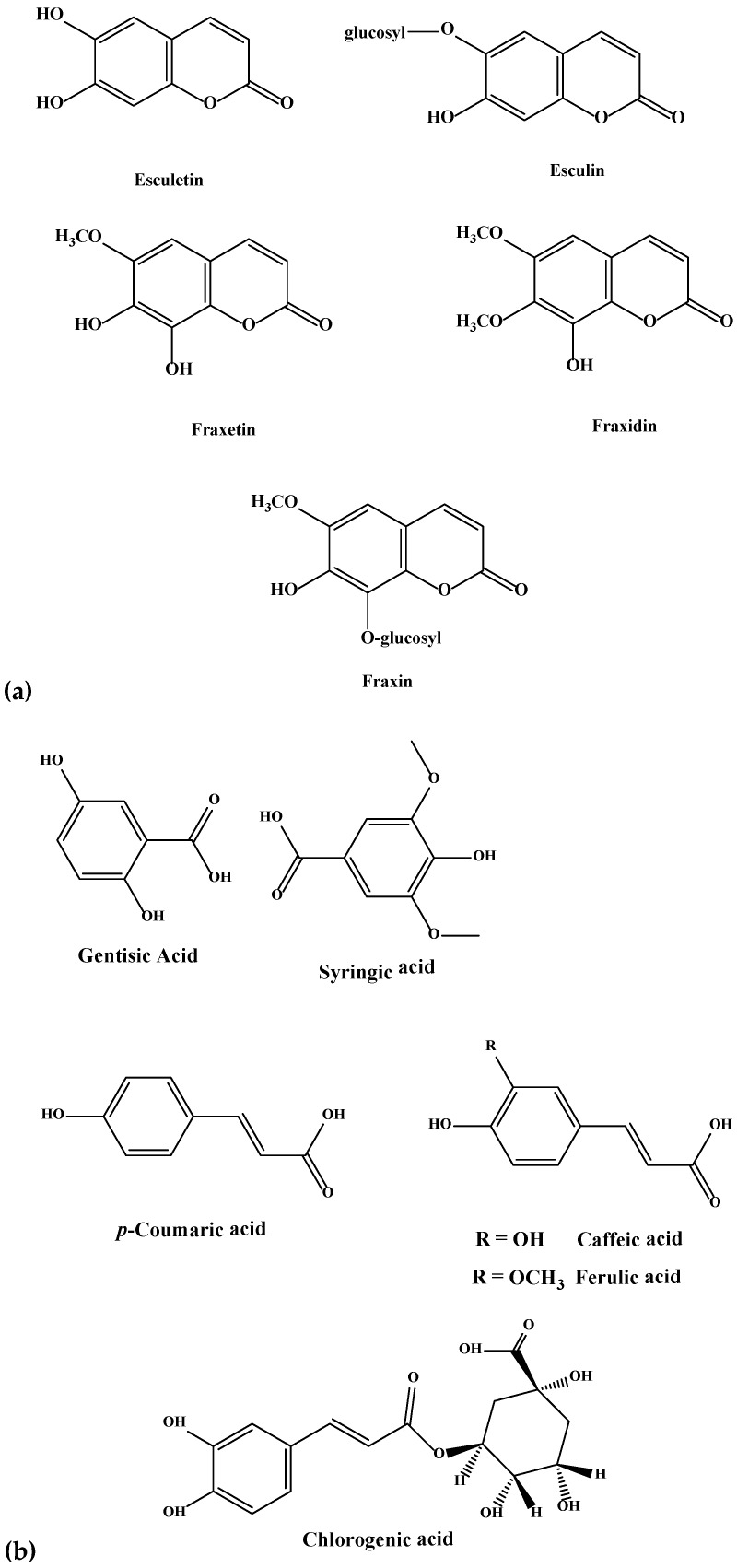

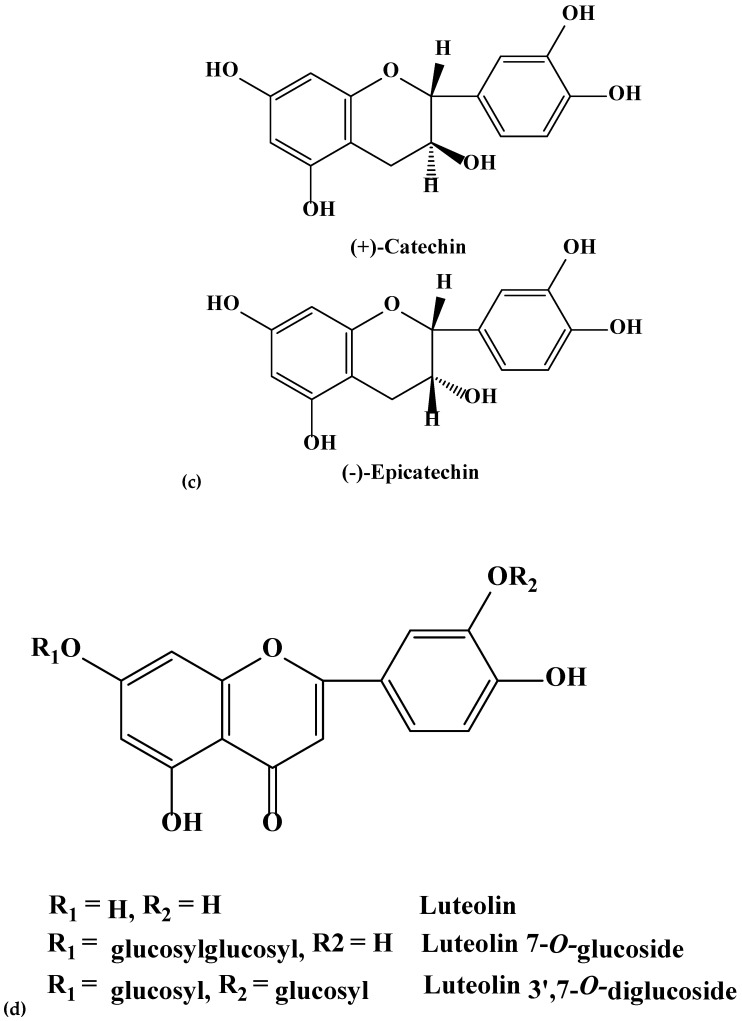

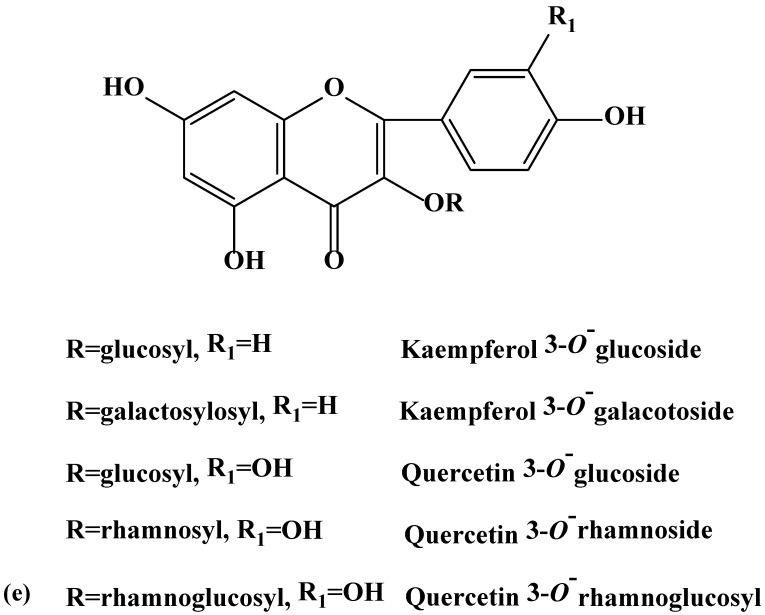
(**a**–**e**). Chemical structures of coumarins (**a**), phenolics (**b**), flavanols (**c**), flavones (**d**), and flavonols (**e**) isolated and purified from green and blues ashes.

Because of the intricate nature of the foliar chemistry of the genus *Fraxinus*, the use of HPLC-MS/MS and autonomous untargeted metabolomics provided the necessary means for investigating the issue. In this study, hydroxycoumarin, phenolic, flavonol, flavanol, flavone and flavanone variations in the leaves of the six *Fraxinus* spp. (green, Manchurian, blue, black, pumpkin and white ashes) compounds were identified using HPLC-MS/MS (Figure 1). A list of 26 compounds and their characterization based on compound classes are presented in Table 1. Spectral analysis and chromatography with standards were used for compound identification. In addition, NMR spectroscopy was employed for the structural elucidation of the isolated compounds. Chemical structures of some of the major compounds identified in this study can be appreciated from the Figure 2. A complete list of the compounds (**1**–**26**) with confirmed identities by NMR is presented in Table 1.

Six hydroxycoumarins have been identified (Table 1) from blue, black and Manchurian ashes in varying amounts, as indicated by percentage peak areas. Esculin was identified in 3-fold high concentration from blue and black ashes in contrast to Manchurian ash. High levels of esculin have also been reported from blue ash [18]. Esculetin was identified in 4-fold high concentration in Manchurian ash in contrast to blue and black ashes. In addition, the compound fraxin was identified in high amounts (8.4 and 1.3-fold, respectively) in Manchurian ash than in blue and black ashes. Similarly, high concentrations of fraxin were reported from Manchurian ash [18]. Fraxetin was identified in high amounts (2.0 and 1.4-fold, respectively) from Manchurian ash in comparison with blue and black ashes. In a previous study, fraxetin was only reported from black ash [18]. High amounts (9 and 3-fold, respectively) of fraxidin was identified from blue ash in contrast to black and Manchurian ashes. In addition, scopoletin was identified in significantly higher concentrations (2.0 5.0, 77 and 86-fold, respectively) from blue ash in comparison to Manchurian, black, pumpkin and white ashes. Scopoletin has been documented as an effective anti-feeding compound against the sunflower beetle and termites [20,21]. Coumarins have also been well documented as contributing to the resistance factor against EAB. In a similar vein, higher concentrations of coumarins were identified from the phloem tissue of Manchurian ash in comparison to black and European ashes [14,15], suggesting these compounds could potentially act as determinants of resistance. In contrast, Whitehill and co-workers reported contrasting results and documented the presence of coumarins from the phloem of black and European ashes in amounts, comparable to or higher than Manchurian ash, implying they are not the determinants of resistance [18].

During this investigation, phenolics and phenolic acids were identified from the leaves of six *Fraxinus* spp. (Table 1) in varying amounts. Amongst phenolics, caffeic, ferulic and *p*-coumaric acids were reported from *F. americana* and *F. excelsior* [22,23]. Furthermore, we identified additional flavonoids and flavonoid glycosides from the six species of *Fraxinus.* Flavones and flavonols have been documented as characteristic compounds for the genus *Fraxinus* [12]. Kaempferol and kaempferol glycosides were identified (Table 1) with varying levels from the leaves of the six *Fraxinus* spp. Similarly, three flavonols, kaempferol-3-*O*-β-d-glucopyranoside, kaempferol-3-*O*-α-l-rhamnopyranosyl-(1→6)-β-d-glucopyranoside and quercetin-3-*O*-α-l-rhamnopyranosyl-(1→6)-β-d-glucopyranoside were identified from the leaves of *F. oxycarba* [24]. Furthermore, kaempferol-3-*O*-rutinoside was also identified from the leaves of *Fraxinus angustifolia* (Vahl) [25].

Kaempferol 3-*O*-(6-*O*-α-l-rhamnopyranosyl-β-d-galactopyranoside), kaempferol 3-*O*-(6-*O*-α-l-rhamnopyranosyl-β-d-glucopyranoside), and kaempferol 7-*O*-(2-*O*-α-l-rhamnopyranosyl-β-d-glucopyranoside) were previously reported from *F. americana* [26]. Kaempferol glycoside and quercetin have also been reported from *F. excelsior* [27]. On the other hand, kaempferol galactoside was identified from the phloem of *F. excelsior* [18]. Isoquercetrin (quercetin 3-*O*-glucoside) and rutin (quercetin 3-*O*-α-l-rhamnopyranosyl-1-6-glucopyranose) have been identified from the leaves of *Fraxinus angustifolia* (Vahl) [25]. Interestingly, we identified the flavanone, naringenin not previously reported from the green ash. Phenolic compounds, a characteristics of the genus *Fraxinus* may have the potential to act as feeding deterrents, toxins and digestion inhibitors [21,28,29].

### 2.2. Metabolomic Analysis of Fraxinus spp.

**Figure 3:**
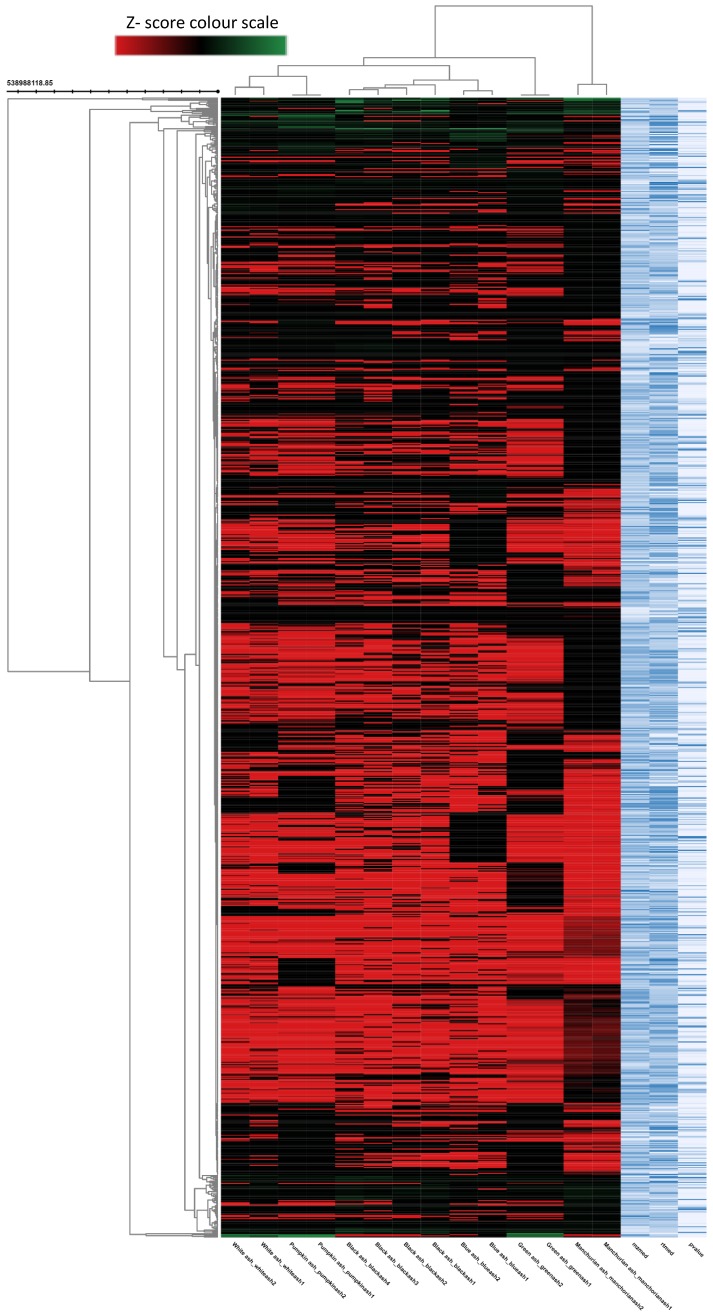
The heat map (dendrogram view) revealing the comparison of metabolite features between the different *Fraxinus* spp. (blue, Manchurian, black, green, pumpkin, and white ashes). In dendrogram, each row represents a metabolite feature and each column represents ash species. Metabolite features, the level of which varies significantly (*p* < 0.05) between different species are projected on the heat map and were used for sample clustering. The row Z score (scaled expression) value of each feature is plotted in red (high abundance) to green color (low abundance) scale.

To better understand the complexity of foliar chemistry in the six *Fraxinus* spp. (green, Manchurian, blue, black, pumpkin and white ashes), the metabolomics approach was adopted. In the past, metabolomics received little attention regarding the genus *Fraxinus*, and only two investigations focused on *F. excelsior* [30,31]. During metabolomic analysis, the autonomous untargeted metabolomics approach provided a global picture of the metabolites from the leaves of six *Fraxinus* spp. The multigroup comparison (Figure 3) revealed in the heat map (dendrogram), demonstrated that the six species of the genus *Fraxinus* clustered into six groups, implying the influence of metabolite features of each species with high clustering coefficient. The untargeted metabolic profiling and metadata analysis resulted in the detection of 1000 metabolite features (*p* < 0.00001) from the six *Fraxinus* spp. The heat map is significant in that it has the unique ability to identify clusters of samples with similar metabolic patterns and discriminating metabolites, which can facilitate sample clustering [32]. The interactive metabolic cloud plot generated (data not presented) during the multi-group analysis revealed that the majority of the metabolite features (*p* < 0.00001) were upregulated in Manchurian ash in contrast to other species. The metabolomic cloud map allowed the detection of metabolite features (based on retention times and *m*/*z* ratios), whose levels differ between different groups (*p* < 0.00001). Overall, the autonomous untargeted metabolomic approach of the XCMS bioinformatics platform provided an efficient means of High-throughput metabolic profiling and metadata analyses of the six species of the *Fraxinus* for comparative analysis.

**Figure 4:**
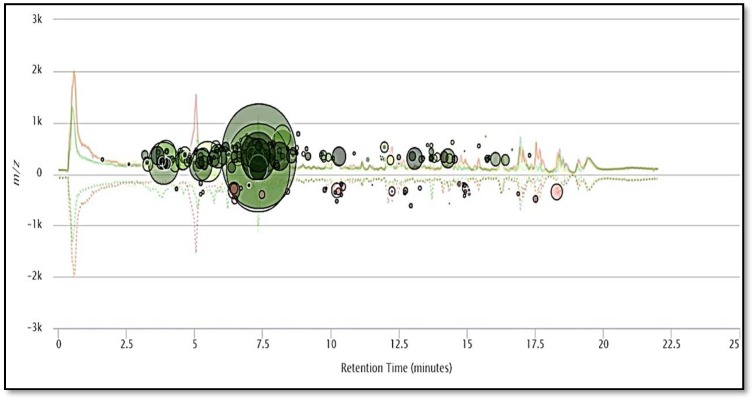
The metabolomic cloud map generated after paired untargeted metabolomics, showing the up- and- down regulated metabolites between blue and Manchurian ashes. The metabolic cloud map revealed a total of 247 metabolite features (*p* < 0.01) between blue and Manchurian ashes. The green circles represent upregulated metabolite features in Manchurian ash, whereas brown circles represent down regulated metabolite feature in blue ash. The cloud map was generated using XCMS bioinformatics platform for metabolomics.

During metabolomic analysis we specifically focused on the comparative analysis of the foliar chemistry between blue and Manchurian ashes, since these two species have different levels of resistance against EAB. Paired metabolomics analysis was conducted between blue and Manchurian ashes using the XCMS bioinformatics platform to delineate the diversity of putative defensive metabolites that may play a role in providing resistance against EAB. The cloud map (Figure 4) generated after paired comparison between blue and Manchurian ashes revealed the identification of 247 metabolite feature (*p* < 0.01). Metabolomic analysis further revealed the presence of compounds, with the majority of them belonging to phenolics, coumarins, lignans and secoiridoids, which are characteristic features of the genus *Fraxinus*. A total of 35 putative compounds were identified from blue and Manchurians ashes (Table 2), most of these compounds were upregulated in Manchurian ash. High-throughput metabolomic data analysis demonstrated the putative identification of secoiridoid compounds, ligstroside and oleuropin identified in significantly higher concentrations (37 and 58-fold) in Manchurian ash. Secoiridoids have biological activity against gram-positive and gram-negative bacteria, herbivores and have anti-inflammatory effects [33,34,35]. Similarly, eight secoiridoid glucosides were reported from the leaves of *F. oxycarba* [24], especially oleuropein, ligstroside, 10-hydroxyoleuropein and 10-hydroxyligstroside. Oleuropein and ligstroside have been documented from *Fraxinus excelsior* [12] and in foliage and phloem tissues of the *F. americana*, *F. pennsylvanica*, *F. mandshurica* and *F. exciclor* [14,18,36]. In addition, secoiridoids, oleuropein and ligstroside have been reported from the leaves of *F. angustifolia* (Vahl) [25].

In this study, paired untargeted metabolomics also helped identify lignans from blue and Manchurian ashes. Matairesinol, pinoresinol glucoside and 8-hydroxypinoresinol-4-glucoside were identified in significantly higher concentrations (7.0, 122 and 34-fold, respectively) in Manchurian ash in contrast to blue ash. Previous investigation reported the presence of three lignans, (+)-pinoresenol-4′-*O*-β-d-glucopyranoside, (+)-fraxiresinol-1-*O*-β-d-glucopyranoside and (+)-1-hydroxypinoresinol-4′-*O*-β-d-glucopyranoside from the leaves of *F. oxycarba* [24]. Pinoresinol, 8-hydroxypinoresinol, fraxiresinol, 8-hydroxysyringaresinol, pinoresinol-4-*O*-β-d-glucopyranoside and 8-hydroxypinoresinol-4-*O*-β-d-glucopyranoside have been reported from *Fraxinus* spp., including *F. mandshurica* and *F. mandshurica* var. japonica [12,17,37,38].

Higher constitutive expressions of an enzyme (phenylcoumaranbenzylic ether reductase) was identified from Manchurian ash that is involved in lignan biosynthesis. Furthermore, this study identified pinoresinol dihexoside unique to Manchurian ash and reported pinoresinol related compounds in higher amounts than green, white and black ashes [18]. Pinoresinol dihexoside and pinoresinol has been shown to have antifeeding and growth inhibiting properties against several insects [39,40,41]. Metabolomic analysis also showed the presence of an iridoid glycoside, verbenalin from blue and Manchurians ashes. Verbenalin was identified highly upregulated (64-fold) in Manchurian ash in comparison with blue ash. Similarly, an iridoid glucoside, 7-epi-7-*O*-(*E*)-caffeoylloganic acid have been identified from the dried leaves of *Fraxinus griffithii* [42]. Recently, putative iridoid glycosides were reported from the leaves of a European common ash, *Fraxinus excelsior*, which is well known for its anti-herbivory terpenoid derivatives [36]. Interestingly enough, Sambles and co-workers [36] reported reduced levels of the iridoid glycosides in tolerant ash, *Fraxinus excelsior*. Metabolomics also revealed the identification of the coumarin, fraxinol, in significant higher concentrations (9 fold) in Manchurian rather than blue ash. Coumarins have been well documented as the hallmark of blue and Manchurian ashes and as determinants of resistance against EAB. Metabolomic analysis revealed sesquiterpenoids (ar-artemisene, ar-turmerone and clovanediol diacetate), a diterpene (totarol) and a sesquiterpene lactone (parthenolide) from the blue and Manchurian ashes (Table 2), not documented in previous studies [14,15,18,19].Apart from the reported metabolites upregulated in Manchurian ash, putative up-regulated compounds have also been identified from blue ash (e.g., dicyclomine, aphidicolin, parthenolide, famciclovir, ar-turmerone and myriocin) that are endemic to North America and have been colonized by EAB at lower levels than other North American ashes [7]. The distinct foliar profile of blue ash observed in this investigation may contribute to its constitutive resistance in comparison to other North American ashes.

### 2.3. System Biology

**Figure 5:**
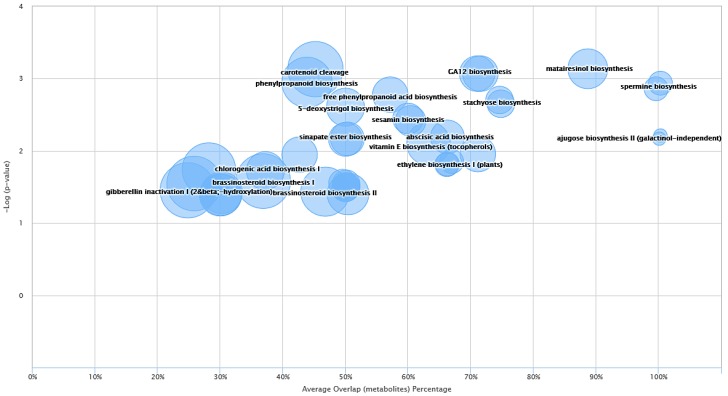
Metabolic cloud plot depicting the predictive pathway analysis between blue and Manchurian ashes. Each pathway is represented by a circle, the x-axis in the cloud plot represents the percentage of metabolites overlap with in the pathway. The y-axis in the pathway represents the increased pathway significance. The radius of each circle is proportional to the total number of metabolites identified in that pathway. The cloud plot of dysregulated metabolites was generated (*p* < 0.05) using XCMS bioinformatics platform.

In order to expand further on the analysis and provide meaningful information about the metabolites identified from blue and Manchurian ashes, system biology analysis guided by XCMS metabolomics was also conducted. Some of the interesting dysregulated pathways identified between blue and Manchurian ashes can be observed in Figure 5. The top three dysregulated pathways identified in this study included, matairesinol biosynthesis (*p* < 0.0007), sinapate and ferulate biosynthesis (*p* < 0.0008), and GA12 biosynthesis (*p* < 0.0008) with an average overlap of metabolites, 89.0%, 71.4% and 71.0% between blue and Manchurian ashes, respectively. The data also suggest that even in dysregulated pathways with high average metabolite overlap (e.g., matairesoniol), the pathways that supply precursor metabolites (e.g., phenylpropanoid, *p* < 0.002) have less metabolite overlap (57%) between the blue and Manchurian ashes. These findings suggest that the dysregulated metabolic pathways operate differently in the blue and Manchurian ashes, and hence resulted in different regulation of metabolites between these two ashes.

### 2.4. Principal Component Analysis (PCA)

**Figure 6:**
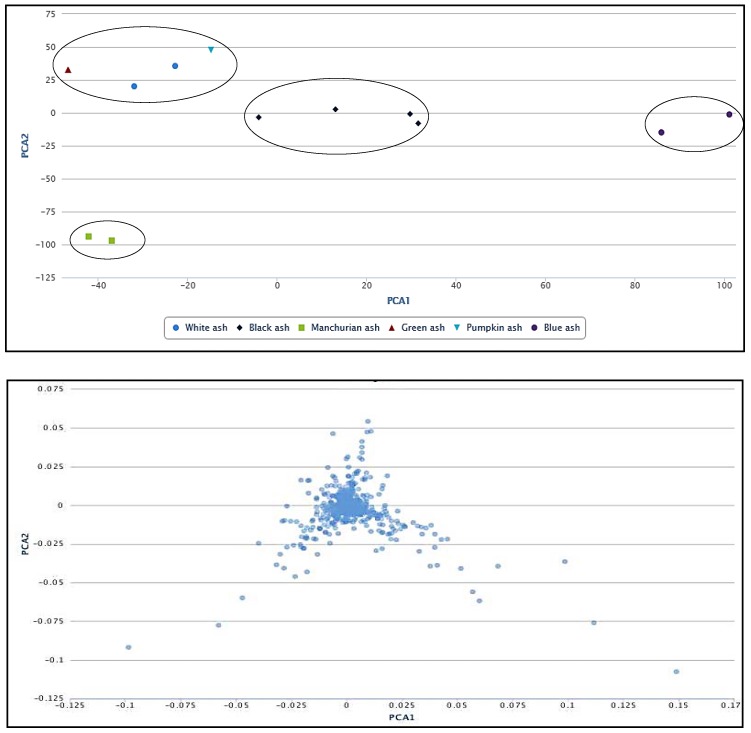
Principal component analysis (PCA; top panel), indicating the correlations between white, black, Manchurian, green, pumpkin and blue ashes. Loading plot (bottom panel) implying the relationship between the metabolite features that relate to the sample grouping. PCA and loading plots were generated using XCMS bioinformatics platform for metabolomics. Four clusters were observed on the PCA plot. Green, white and pumpkin ashes were clustered in section, *Meliodes*. Two clusters were observed for the section *Fraxinus* (referred as *Fraxinus* a and b), occupied by black and Manchurian ashes. Blue ash was clustered in a section, *Dipetelae*.

To show the relationship among the six *Fraxinus* spp. under investigation, we used principal component analysis (PCA) that is considered one of the most widely used multivariate analysis tools in untargeted metabolic profiling and/or fingerprinting [43]. The PCA revealed the relationship between different species, which makes use of covariance or correlations among variables or metabolite features. Four main clusters were observed on the PCA plot (Figure 6), implying the influence of metabolite features in the clustering. The corresponding loading plot indicating the correlations between metabolites and their relationship to the sample grouping can be appreciated from Figure 6. We observed blue ash in a separate cluster (*Dipetalae*), implying its distinct chemistry and phylogeny (Figure 6). The green, white and pumpkin ashes clustered in a separate group (*Melioides*). Interestingly, we identified two separate clusters of section *Fraxinus* (referred as *Fraxinus* a and b), each occupied by black and Manchurian ashes. These data imply that metabolite features have more influence in the clustering of these two species, rather than their phylogenetic relationship. In contrast, high convergence in the phenolic profiles between black, Manchurian and European ashes has been observed in a study that placed them in the same taxonomic section [18]. It is important to mention that the PCA plot presented in the current study is based on 1000 metabolite features (*p* < 0.00001). Furthermore, the clonally propagated cultivar studied earlier [18], may not represent the open pollinated species of the genus *Fraxinus* in its true sense.

## 3. Methods and Materials

### 3.1. Plants

*Fraxinus pennsylvanica* var. subintegerrima (Vahl) Fern. (Green ash), *F. americana* L. (White ash), *F. profunda* (Bush) Bush. (Pumpkin ash), *F. quadrangulata* Michx. (Blue ash) and *F. nigra* Marsh. (Black ash) leaves were collected in June, July and August 2004–2006 from Sault Ste. Marie (lat. 46°30′ N, long. 84°20′ W), Sombra, (lat. 42°42′ N, long. 82°29′ W), Rondeau Provincial Park (lat. 42°17′ N, long. 81°52′ W), Chatham (lat. 42°24′ N, long. 82°11′ W), Cambridge (lat. 43°23′ N, long. 80°19′ W) and Windsor (lat. 42°18′ N, long. 83°01′ W), Ontario, Canada. Plants of *F. mandshurica* (Manchurian ash) were locally grown in Sault Ste. Marie and the leaves collected for extraction. Pressed voucher specimens of all species are deposited in the Natural Products Laboratory, Canadian Forest Service-Sault Ste. Marie herbarium.

### 3.2. Extractions

Bulk extract. Fresh or previously frozen leaf material was extracted at room temperature in two sequential steps: First, by steeping for 72 h in 70% EtOH (aqueous), followed by maceration of the foliage, decanting and filtering of the extract; secondly, by steeping the remaining residue for an additional 24 h employing the same solvent system; however, using 30–50% of the initial volume. The combined and filtered aqueous ethanolic extracts were evaporated under reduced pressure, frozen and subjected to freeze drying to obtain freeze-dried extracts.

Green Ash, MacAlpine Site, Chatham, ON, 2.18 kg/24 L 70% EtOH, 118.01 g; White Ash, Bellevue Park/GLFC, Sault Ste. Marie, ON, 3.45 kg/36 L 70% EtOH, 393.38 g; Pumpkin Ash, Kopegaron Woods Conservation Area, Leamington, ON, 0.74 kg/20 L 70% EtOH, 59.9 g; Blue Ash, D’Arcy McKeough Conservation Area, Sombra, ON, 5.62 kg/36 L 70% EtOH, 233.50 g; Black Ash, Fish Hatchery Rd. Sault Ste. Marie, ON, 4.76 kg/38 L 70% EtOH, 377.22 g and Manchurian Ash, GLFC Grounds, Sault Ste. Marie, ON, 0.04 kg/3 L 70% EtOH; 5.9 g.

### 3.3. Fractionation

Individual samples of green and blue ash freeze-dried extracts (100 g) were each mixed with 200 g of polyvinylpolypyrrolidone (PVPP) powder (Sigma). The homogeneous mixture of either sample was then placed on top of a PVPP packed 4 L Buchner funnel. Elution of individual extracts was carried out at a slow rate initially with 5 L of water followed by 5 L aliquots of increasing concentrations (20%, 50%, 70% and 100%) of aqueous ethanol to produce five fractions. Each fraction was concentrated under vacuum and chromatographed one dimensionally on Whatman No.1 chromatography paper using one or more solvent systems: BAW (*n*-butanol-acetic acid-water, 4:1:5, upper phase), water, or acetic acid-water (15:85). Bands detected by absorbance/fluorescence under short wave light (254 nm) and long wave light (366 nm) were identified, cut and eluted with methanol. The samples were evaporated to dryness, dissolved in a minimum volume of methanol, and placed onto a PVPP column from which they were eluted sequentially with the following solvent systems: (i) CH_2_Cl_2_-EtOH-MeCOEt-Me_2_CO (1:1:1:1), (ii) EtOH-MeCOEt-Me_2_CO-H_2_O (1:1:1:1) and (iii) EtOH-H_2_O (1:1). Final clean up and purification of the compounds was achieved on a Sephadex LH-20 column (1 cm × 50 cm), using methanol as the eluting solvent.

### 3.4. Identification of Isolated Compounds

UV spectra were recorded on a UV-Vis Beckman DU series 800 spectrophotometer (Brea, California, CA, USA). ^1^H-NMR and ^13^C-NMR spectra were recorded on a Bruker AMX-400 spectrometer (Billerica, MA, USA), at 500 MHz and 125 MHz respectively; samples were dissolved in DMSO-*d*_6_ with TMS as an internal standard. Structures of purified compounds were determined according to standard methods [44,45] acid hydrolysis in 2 M and 0.1 M HCl (mild hydrolysis) at 100 °C for 60 min; enzymatic hydrolysis with β-glucosidase (Sigma-Aldrich, St. Louis, MO, USA) using an acetate buffer (pH 5); hydrogen peroxide oxidation; UV spectroscopy; ^1^H-NMR; ^13^C-NMR and FAB-mass spectroscopy (positive and negative), and by comparison with authentic samples where available. The glycosides and aglycones obtained by hydrolysis of isolated compounds were identified by co-chromatography with authentic samples (Sigma-Aldrich, St. Louis, MO, USA; Apin Chemicals LTD., Abingdon, OX., United Kingdom and Extrasynthese, Genay Cedex, France) using PC, TLC, HPLC and UPLC. Sugars released by hydrolysis were identified by PC and TLC using available standards.

### 3.5. Preparation of Extracts for HPLC-MS/MS

The crude freeze-dried extracts were dissolved in Acetonitrile (AcN) (LC/MS grade, Fisher Scientific, Ottawa, ON, Canada) at concentrations of 10 mg/mL and were filtered through 0.22 µm GHP filters (Acrodisc Syringe Filters, Pall Corporation, Quebec, QC, Canada) prior to HPLC-MS/MS analysis.

### 3.6. HPLC-MS/MS (ORBITRAP)

High performance liquid chromatography was performed using a Thermo Scientific LTQ Orbitrap Discovery (MS 2.5.5) equipped with an Autosampler Accela AS 2.2.1, and pump 1.04.05. The instrument was equipped with a Syncronis C18, Thermo Scientific column: 50 mm length, 2.1 mm I.D., and 1.7 µm particle size that was operating at normal room temperature. Injection volumes were 10 µL. A gradient technique was employed in this study with a flow rate of 0.2 mL/min. Solvent A was composed of acetonitrile (AcN) acidified with 0.1 vol % of formic acid whereas solvent B was composed of water acidified with 0.1 vol % of formic acid. The gradient was programmed as follows: Solvent A: 2 vol %, increased to 10 vol % at 2 min, increased to 25 vol % at 6 min, increased to 50 vol % at 10 min, increased to 75 vol % at 14 min, increased to 95 vol % at 18 min, decreased to 2 vol % at 20 min, followed by 2 min of isocratic elution with 2% of solvent A (total elution time 22 min). The LTQ Orbitrap MS was equipped with an ESI source operating in the positive ionization mode using the following operating parameters: Electrospray voltage of 3.1 kV, sheath gas flow rate of 8 abu (arbitrary units), auxiliary gas flow rate of 1 abu, capillary temperature of 270 °C, capillary voltage set to 49.00 V, tube lens offset at −148.43 V. Instrument calibration was performed externally prior to running each sequence, employing the “Thermo Scientific Pierce LTQ Velos ESI positive ion calibration solutions.” Accurate mass spectra of [M + H]^+^ ions were recorded from 100 to 1000 *m*/*z*, the mass resolution power of the mass analyzer was set to 30,000 (m/∆m) at *m*/*z* 400. Nitrogen gas (purity 99.95%) was used both as sheath gas and auxiliary gas and also served as the co collision gas in the HCD cell and the bath gas in the C-trap. Percentage peak areas of the compounds were calculated using Xcalibur (version 2.2, Thermo Scientific, Waltham, MA, USA) using the peak detection algorithm, ICIS, specifically designed for MS data that has superior peak detection efficiency at low MS signal levels.

### 3.7. Untargeted Metabolomic Analysis

Data were analyzed using multi-group method of XCMS online (https://xcmsonline.scripps.edu) bioinformatics platform [46]. In addition, paired comparison between blue and Manchurians ashes was performed to identify putative up- and- down regulated metabolites. The HPLC-MS/MS raw data files from ORBITRAP (Discovery) were converted to mzXML format using Proteowizard, and were subsequently processed for peak detection, retention time correction, chromatogram alignment, metabolite feature metadata/statistical analysis, and putative identification using METLIN database. The parameter settings for XCMS processing were as follows: centWave for feature detection (∆ *m*/*z* = 15 ppm, minimum peak width = 10 s and maximum peak width = 120 s); obiwarp settings for retention-time correction (prof Step = 1); and parameters for chromatogram alignment, including mzwid = 0.015, minfrac = 0.5 and bw = 5. The relative quantification of metabolite features was based on peak areas [47,48].

## 4. Conclusions

The current study presents the first report on the High-throughput metabolomic analysis of the foliar chemistry of the six *Fraxinus* spp. Phenolics and flavonoids variations from the six *Fraxinus* spp. was identified, not documented in earlier studies. Compounds were purified, and the identities of phenolics and flavonoids were confirmed by NMR. Metabolomic profiling using HPLC-MS/MS in combination with NMR provided chemical differentiation of the six *Fraxinus* spp., hence, an insight on the foliar phytochemical diversity of these plants. Metabolomics provided a useful tool for the rapid identification of metabolites and revealed coumarins, secoiridoids and lignans from blue and Manchurian ashes.

The striking feature of this study, the paired untargeted metabolomic analysis revealed several putative defensive compounds for the first time from blue and Manchurian ashes (Table 2). Interestingly, we identified sesquiterpenoids, a diterpene and a sesquiterpene lactone, not documented earlier from these ashes. The systems biology analysis unique to this investigation suggests the differential regulation of metabolites between blue and Manchurian ashes. Further, paired metabolomics showed majority of the compounds upregulated in Manchurian ash, which may suggest their role in providing resistance, and reflects its co-evolutionary history with *A. planipennis* where they coexist in their native habitat. These data suggest that clear differences exist in the chemical defensive profiles between resistant ash species and its susceptible counterparts in North America. Our study supports the potential role of coumarins, secoiridoids, lignans, sesquiterpenoids, diterpenes and sesquiterpene lactones in providing constitutive resistance to Manchurian ash, hence the synergistic action of the upregulated defensive compounds may provide a mechanism of resistance to Manchurian ash against EAB.

The autonomous untargeted metabolomics provided High-throughput screening and metabolic profiling of phytochemicals from six *Fraxinus* spp. that can provide the groundwork for the future studies. Since, a significant portion (25%) of the ash genome encodes unique or orphan genes [30], metabolomics (especially the systems biology approach) would prove to be a useful tool in order to facilitate the putative identification of metabolites and key metabolic pathways that can help understand and identify gene functions. The metabolomics would prove to be especially useful in the discovery of potential biomarkers for the rapid and early screening of diseased ash trees and resistant ash varieties for the breeding programs.

## Figures and Tables

**Table 1:** HPLC-MS/MS analysis used for the identification of compounds from blue, Manchurian, black, green, pumpkin, and white ashes. Actual mass, M + H, RT, and percentage peak areas among reported compounds.

					*Dipetalae*	*Fraxinus*		*Melioides*		
No	Compound Name									
		Actual Mass	M + H	RT	Blue Ash	Manchurian Ash	Black Ash	Green Ash	Pumpkin Ash	White Ash
	***Coumarins***									
**1**	Esculin	340.284	341.0857	2.8	0.31	0.12	0.37	N.D.	N.D.	N.D.
**2**	Esculetin	178.143	179.0335	3.55	0.07	0.32	0.08	N.D.	N.D.	N.D.
**3**	Fraxetin	208.169	209.0439	4.2	0.11	0.21	0.15	N.D.	N.D.	N.D.
**4**	Fraxidin	222.196	223.0595	5.64	0.46	0.17	0.05	N.D.	N.D.	N.D.
**5**	Fraxin	370.310	371.0962	5.7	0.05	0.42	0.32	N.D.	N.D.	N.D.
**6**	Scopoletin	192.170	193.0492	5.0	6.89	3.09	1.37	N.D.	0.09	0.08
	***Phenolics/Phenolic Acids***									
**7**	Chlorogenic acid	354.311	355.1018	3.5	0.08	0.13	0.07	0.04	0.06	0.17
**8**	Caffeic acid	180.159	181.0496	3.7	0.08	0.58	N.D.	N.D.	N.D.	N.D.
**9**	Syringic acid	198.174	199.0601	3.9	0.04	0.07	N.D.	N.D.	N.D.	N.D.
**10**	Ferulic acid	194.186	195.0651	5.2	0.84	N.D.	N.D.	N.D.	N.D.	N.D.
**11**	*p*-Coumaric acid	164.160	165.0546	4.8	0.79	0.1	0.08	N.D.	0.04	0.04
**12**	Gentisic acid	154.121	155.0338	5.74	0.24	N.D.	N.D.	N.D.	N.D.	N.D.
	***Flavanols***									
**13**	(+) Catechin	290.271	291.0859	10.0	0.15	0.04	0.02	0.18	0.01	0.03
**14**	(−)-Epicatechin	290.271	291.0859	15.3	0.09	0.12	0.06	0.15	0.01	0.09
	***Flavones***									
**15**	Apigenin	270.240	271.0597	8.6	N.D.	N.D.	N.D.	0.68	0.18	N.D.
**16**	Luteolin	286.239	287.0545	7.7	N.D.	N.D.	N.D.	0.1	0.05	N.D.
**17**	Luteolin-7-*O*-glucoside	448.380	449.1068	5.5	N.D.	0.89	N.D.	1.4	0.17	0.16
**18**	Luteolin-3’,7-*O*-β-d-diglucoside	610.518	611.1589	4.9	N.D.	N.D.	N.D.	0.04	0.02	0.21
	***Flavonols***									
**19**	Quercetin-3-*O*-β-d-glucoside	464.379	465.1018	5.6	0.57	0.35	0.1	0.37	0.11	0.84
**20**	Quercetin-3-*O*-β-d-galactoside	464.379	465.1018	5.48	N.D.	0.64	0.28	0.03	0.1	0.04
**21**	Quercetin-3-*O*-β-l-rhamnoside	448.380	449.1068	6.14	0.14	1.1	0.13	3.32	0.68	1.0
**22**	Quercetin-3-*O*-rhamnoglucoside	610.521	611.1589	5.4	0.34	0.15	0.2	N.D.	0.34	0.86
**23**	Kaempferol-3-*O*-β-d-glucoside	448.380	449.1070	6.08	0.26	0.07	0.1	N.D.	N.D.	0.13
**24**	Kaempferol-3-*O*-rhamnoglucoside	594.522	595.1643	5.9	N.D.	0.3	0.32	N.D.	0.3	0.2
**25**	Kaempferol-2-*B*-coumaryl glucoside	594.525	595.1432	7.8	N.D.	N.D.	N.D.	N.D.	0.06	N.D.
	***Flavanone***									
**26**	Naringenin	272.257	273.0752	8.4	N.D.	N.D.	N.D.	0.51	N.D.	N.D.

N.D. Not detected. Compounds (**1–26**) were identified using authentic standards. Identities of the compounds (**1–26**) were confirmed by NMR.

**Table 2:** Metabolomic analysis (paired untargeted metabolomics) revealing the putative identification of up- and- down regulated metabolites in Manchurian ash in comparison to Blue ash. Formula, M + H, RT, fold change, up- and- down regulation of metabolites, and *p* value (Welch’s *t* test) among reported compounds.

No	Compound Name	Formula	Actual Mass	M + H	RT	Fold Change	Up/Down	*p* Value
**1**	Dicyclomine	C_19_H_35_NO_2_	309.487	310.2736	9.78	50	DOWN	0.00047
**2**	*N*-hydroxyamphetamine	C_9_H_13_NO	151.205	152.1069	8.03	13	UP	0.00055
**3**	Norethynodrel	C_20_H_26_O_2_	298.419	299.2002	15.8	30	UP	0.00056
**4**	4-(3,4-Difluorophenyl) piperidine	C_11_H_13_F_2_N	197.101	198.1086	10.38	30	DOWN	0.00059
**5**	Erythronolide A	C_17_H_30_N_4_	418.527	419.2685	8.7	18	UP	0.00067
**6**	Phenol, 4-amino-2,6-*bis*(1-piperidinylmethyl)-	C_18_H_29_N_3_O	303.450	304.2314	14.31	32	UP	0.00082
**7**	Butyronitrile, 2,2-diphenyl-4-(4-(3-hydroxymethyl-2-oxo-1-benzimidaizolinyl) piperidino)-	C_29_H_30_N_4_O_2_	466.585	467.5260	9.1	28	UP	0.00094
**8**	Pinoresinol glucoside	C_26_H_32_O_11_	520.525	521.2012	6.39	122	UP	0.01517
**9**	8-hydroxypinoresinol 4-glucoside	C_26_H_32_O_12_	536.530	537.1959	4.75	34	UP	0.01435
**10**	Mandarone A	C_20_H_26_O_3_	314.425	315.1953	8.31	7.0	UP	0.02711
**11**	*N,N*-Dicyclohexyl-2,3,4,5,6-pentahydroxyhexanamide	C_18_H_33_NO_6_	359.463	360.2377	10.16	15	DOWN	0.00097
**12**	2,2’-Disulfanediylbis[Nt-(hepta-1,6-dien-4-yl) aniline]	C_26_H_32_N_2_S_2_	436.200	437.2079	13.6	58	UP	0.00098
**13**	Matairesinol	C_20_H_22_O_6_	358.141	359.1482	7.9	6.7	UP	0.00550
**14**	Benzamide, *N*-(2-(diethylamino) ethyl)-4-((1-oxopropyl) amino)-, monohydrochloride	C_16_H_26_ClN_3_O_2_	327.171	328.2055	12.99	156	UP	0.00112
**15**	beta-Ionone	C_13_H_20_O	192.151	193.1585	8.0	12	UP	0.00113
**16**	Ligstroside	C_25_H_32_O_12_	524.519	525.2226	7.34	37	UP	0.00118
**17**	2-Methoxy-1-(phenylselanyl) decan-3-ol	C_17_H_28_O_2_Se C_17_H_28_O_2_Se	344.125	345.1328	7.36	56	UP	0.00130
**18**	Totarol	C_20_H_30_O	286.229	287.2366	16.04	264	UP	0.00144
**19**	Clovanediol diacetate	C_19_H_30_O_4_	322.214	323.2212	12.74	29	DOWN	0.00147
**20**	Mulberrofuran T	C_44_H_44_O_9_ C_44_H_44_O_9_	716.298	717.3144	8.8	24	UP	0.00149
**21**	ar-Artemisene	C_20_H_30_	270.234	271.2416	16.41	109	UP	0.00171
**22**	11,12-dihydroxy arachidic acid	C_20_H_40_O_4_	344.292	345.2994	8.22	20	UP	0.00174
**23**	1-Docosylpyridin-1-ium bromide	C_27_H_50_BrN	467.312	468.3465	17.52	57	DOWN	0.00221
**24**	Aphidicolin	C_20_H_34_O_4_	338.245	339.2525	10.29	35	DOWN	0.00236
**25**	Parthenolide	C_15_H_20_O_3_	248.141	249.1481	10.46	20	DOWN	0.00291
**26**	Pentaquine	C_18_H_27_N_3_O	301.215	302.2156	9.74	56	UP	0.00293
**27**	Famciclovir	C_14_H_19_N_5_O_4_	321.143	322.1773	8.42	24	DOWN	0.00302
**28**	ar-Turmerone	C_15_H_20_O	216.151	217.1583	14.92	22	DOWN	0.00309
**29**	Myriocin	C_21_H_39_NO_6_	401.277	402.2846	10.4	20	DOWN	0.00439
**30**	Coniferin	C_16_H_22_O_8_	342.131	343.1384	4.94	14	UP	0.07002
**31**	Isosyringinoside	C_23_H_34_O_14_	534.194	535.1834	6.36	54	UP	0.03068
**32**	Oleuropein	C_25_H_32_O_13_	540.184	541.2175	6.58	58	UP	0.00360
**33**	Hydroxytyrosol 1-*O*-glucoside	C_14_H_20_O_8_	316.115	317.1492	1.25	16	UP	0.03709
**34**	Verbenalin	C_17_H_24_O_10_	388.136	389.1437	3.11	64	UP	0.01169
**35**	Fraxinol	C_11_H_10_O_5_	222.052	223.0599	5.2	9.3	UP	0.00060
